# The Age-Related Perfusion Pattern Measured With Arterial Spin Labeling MRI in Healthy Subjects

**DOI:** 10.3389/fnagi.2018.00214

**Published:** 2018-07-17

**Authors:** Nan Zhang, Marc L. Gordon, Yilong Ma, Bradley Chi, Jesus J. Gomar, Shichun Peng, Peter B. Kingsley, David Eidelberg, Terry E. Goldberg

**Affiliations:** ^1^The Litwin-Zucker Research Center, The Feinstein Institute for Medical Research, Northwell Health, Manhasset, NY, United States; ^2^Department of Neurology, Tianjin Neurological Institute, Tianjin Medical University General Hospital, Tianjin, China; ^3^Donald and Barbara Zucker School of Medicine at Hofstra-Northwell, Hofstra University, Hepstead, NY, United States; ^4^Center for Neurosciences, The Feinstein Institute for Medical Research, Northwell Health, Manhasset, NY, United States; ^5^Department of Radiology, North Shore University Hospital, Northwell Health, Manhasset, NY, United States

**Keywords:** arterial spin labeling, cerebral blood flow, aging, principal component analysis, scaled subprofile model

## Abstract

**Aim:** To analyze age-related cerebral blood flow (CBF) using arterial spin labeling (ASL) MRI in healthy subjects with multivariate principal component analysis (PCA).

**Methods:** 50 healthy subjects (mean age 45.8 ± 18.5 years, range 21–85) had 3D structural MRI and pseudo-continuous ASL MRI at resting state. The relationship between CBF and age was examined with voxel-based univariate analysis using multiple regression and two-sample *t*-test (median age 41.8 years as a cut-off). An age-related CBF pattern was identified using multivariate PCA.

**Results:** Age correlated negatively with CBF especially anteriorly and in the cerebellum. After adjusting by global value, CBF was relatively decreased with aging in certain regions and relatively increased in others. The age-related CBF pattern showed relative reductions in frontal and parietal areas and cerebellum, and covarying increases in temporal and occipital areas. Subject scores of this pattern correlated negatively with age (*R*^2^ = 0.588; *P* < 0.001) and discriminated between the older and younger subgroups (*P* < 0.001).

**Conclusion:** A distinct age-related CBF pattern can be identified with multivariate PCA using ASL MRI.

## Introduction

Cerebral blood flow (CBF) refers to the rate of delivery of arterial blood to the capillary bed in brain tissue and is tightly correlated with neuronal activity and metabolism in healthy subjects. Resting state CBF is less susceptible to intra- or inter-individual differences in behavioral performance compared with task-related measures. In addition, due to the simplicity of resting state CBF, it has superior feasibility in investigating older subjects and patients with cognitive impairment. Arterial spin labeling (ASL) is a novel magnetic resonance imaging (MRI) technique to quantitatively measure CBF using arterial blood water as an endogenous tracer (Alsop et al., 2015). In contrast with other perfusion measurements, such as H_2_^15^O positron emission tomography (PET), ^99m^Tc-ethylcysteinate dimer single photon emission computed tomography (SPECT) and dynamic contrast-enhanced MRI, ASL has several advantages including (1) no expensive or potentially harmful tracers; (2) non-invasive procedure; (3) stability for tracking CBF changes over time; and (4) short scan times for repeat imaging studies (Wintermark et al., 2005).

Aging is an important risk factor for cerebrovascular disease and neurodegenerative diseases. In order to properly interpret CBF changes in these disease conditions, it is important to understand normal age-related changes in CBF (Zhang et al., 2017). Age-associated CBF reduction in the whole brain and in frontal, temporal and parietal regions has been observed in previous studies using PET, SPECT, ^133^Xe contrast computed tomography and ASL MRI (Pantano et al., 1984; Shaw et al., 1984; Takahashi et al., 2005; Chen et al., 2011). Of note, relatively increased regional CBF, especially in temporal lobes, has also been detected with ASL in elderly compared to younger subjects after accounting for the effect of global CBF (Preibisch et al., 2011). The effect of aging on CBF alteration measured with ASL MRI was previously investigated using univariate analysis; however, aging affects brain systems or networks rather than individual regions. The particular network of age-related CBF changes is still not well established with this imaging modality.

Univariate analysis can detect regionally specific changes in brain function but usually ignores the functional correlations between anatomical structures of interest. Scaled subprofile model (SSM) is a multivariate method based on principal component analysis (PCA) to identify significant spatial covariance patterns in functional brain images. Compared to univariate analysis, this multivariate statistical model is based on the notion that small but biologically relevant signals can be detected in functional brain images after minimizing the large variability that exists across subjects and regions (Eidelberg, 2009). It has been reported with SSM analysis that the reduced regional metabolism measured with fluorodeoxyglucose (FDG) PET showed a gradient from frontal to parieto-occipital areas in healthy aging (Moeller et al., 1996). In the current study, we assessed CBF changes with pseudo-continuous ASL (pCASL) at resting state in healthy subjects across a relatively large age span, and sought to identify the age-related CBF pattern for the first time using SSM/PCA of ASL MRI data.

## Materials and Methods

### Participants

Fifty healthy individuals (mean age = 45.76 ± 18.45 years, age range: 21–85, 28 females) were recruited from the Feinstein Institute for Medical Research. All participants were cognitively normal, with Mini-mental State Examination scores ≥ 24 or Mattis Dementia Rating Scale score ≥ 130. All subjects had no history of neurological diseases, mental disorders or brain injury, and refrained from alcohol, caffeine and nicotine for at least 6 h prior to MRI measurement. No infarctions or significant ischemic changes were observed on brain MRI images for any participants. For group comparisons, the subjects were divided into younger and older groups according to a median age of 41.8 years. The mean age of the younger group was 30.46 ± 5.87 years. The mean age of the older group was 61.05 ± 13.17 years. There were no significant differences in sex or educational level between these two groups (**Table 1**). The study was approved by the Northwell Health IRB. Informed consent was obtained from all participants.

**Table 1:** Demographics of all healthy participants.

	Younger group *N* = 25	Older group *N* = 25	*P*
Age, y	30.46 (5.87)	61.05 (13.17)	0.000
Sex, F/M	17/8	11/14	0.087
Education, y	16.60 (2.12)	17.36 (2.27)	0.227
Handedness, R/L/A	23/2/0	19/4/2	0.218

*Age and education are provided as mean (SD). R, right-handed; L, left-handed; A, ambidextrous.*

### MRI Acquisition

All imaging studies were performed on a GE Twinspeed 3-T MRI scanner with HDx technology, using an 8-channel phased array head coil (GE Healthcare, Milwaukee, WI, United States). A T1-weighted 3D inversion recovery spoiled gradient recalled (IR-SPGR) series was first acquired (echo time/repetition time [TE/TR]: 2.996 ms /7.788 ms, inversion time: 450 ms, flip angle: 20°, field of view: 240 mm, voxel size: 0.9375 mm × 0.9375 mm, matrix size: 256 × 256, slice thickness: 1.5 mm, number of slices: 136) to serve as a template for co-registration with ASL imaging data. A stack of spiral, fast spin-echo images were prepared with pCASL and background suppression to measure whole brain perfusion (TE/TR: 9.804 ms/4580 ms, labeling duration: 1525 ms, post labeling delay: 1525 ms, flip angle: 155°, acquisition matrix: 128 × 128, field of view: 240 mm, voxel size: 1.875 mm × 1.875 mm, slice thickness: 4 mm, number of slices: 34, total number of CBF image volume: 1). During the resting state scan of ASL, participants had their ears plugged, and were instructed to keep their eyes closed, not to think of anything in particular, not to fall asleep, and to remain still during each series.

### ASL Data Preprocessing

All images were preprocessed with statistical parametric mapping (SPM12, Institute of Neurology, London, United Kingdom) software running on a Linux computer in MATLAB (Version R2016a; MathWorks, Natick, MA, United States). ASL MRI images were converted into maps of CBF to identify changes in regional CBF. Image preprocessing was conducted as follows: (1) Co-registration: CBF images were registered to structural MRI images; (2) Normalization and Segmentation: the structural images were normalized to the standard Montreal Neurological Institute (MNI) brain template and then segmented into probability maps of gray matter, white matter and cerebrospinal fluid; (3) Normalization and Masking: the CBF images were normalized using the parameters determined from the structural images and multiplied by a binary brain tissue mask only consisting of gray matter and white matter; (4) Smoothing: the normalized CBF images were then smoothed with a 10 mm Gaussian kernel to reduce inter-individual anatomical differences and increase the signal-to-noise ratio.

### Statistical Analysis

#### Univariate Analysis

All data analysis was performed within a gray matter mask (threshold ≥ 0.3) using SPM12 software. The relationship between CBF and age was examined using a multiple regression model with sex as a covariate. We also performed a group comparison to determine the CBF differences between older and younger groups with a two-sample *t*-test model, using sex as a covariate. Coordinates were reported in the standard MNI anatomical space. With a voxel-level peak threshold of *P* < 0.001 (uncorrected) over whole brain regions, we primarily identified clusters > 200 voxels (voxel size = 1.5 mm × 1.5 mm × 1.5 mm) for the analysis of absolute CBF and clusters > 100 voxels after adjusting for global values with ANCOVA. For a stricter criterion, we also highlighted clusters that survived a family wise error correction at *P* < 0.05. Significant regions were localized by Talairach–Daemon software (Research Imaging Center, University of Texas Health Science Center, San Antonio, TX, United States). The SPM maps for CBF with age were overlaid on a standard T1-weighted MRI brain template in stereotaxic space.

To quantify CBF changes in specific regions, we used a 4 mm radius spherical volume of interest centered at the peak voxel of clusters that were significant in the SPM analyses. We then calculated the relative (i.e., globally adjusted) CBF values in all participants with SPM12. The relationship between relative CBF value and age was analyzed with linear regression.

#### Multivariate Analysis

Scaled subprofile model, which is one of the multivariate spatial covariance techniques based on PCA, was applied to assess subject-by-voxel effects on CBF maps in all participants with Scaled Subprofile Model Principal Component Analysis (SSMPCA) toolbox^1^. These procedures produced a set of brain network covariance patterns and corresponding subject scores that reflect the degree to which each subject expresses these network patterns individually. Multiple regression analysis was used to identify the best set of SSM component patterns predicting age. We first tested the significance of the regression model predicting age using subject scores of the first 6 patterns that accounted for >60% of subject × voxel variance in the SSMPCA operation, and subsequently identified a subset of these 6 patterns whose linear combination provided the best correlation between the pattern score and subject age in this sample. These component patterns and their subject scores were linearly combined using the regression parameters to define an age-related pattern and corresponding subject scores. Subject scores were then z-transformed using mean and standard deviation of the whole group. The reliability of the resulting topography was evaluated by using a bootstrapping resample scheme described previously (Habeck et al., 2008). Anatomical localization and display of the age-related pattern were performed using the same procedures as for the SPM maps described above.

To clarify sex effects on CBF and PCA pattern, we also compared the differences in global CBF value and relative CBF values in specific regions, and PCA network score between female subjects and male subjects, using a general linear model with age as a covariate. The relationship between global CBF value, relative CBF values in specific regions, network score and age was further analyzed with linear regression in female subjects and male subjects, respectively.

## Results

### Voxel Based Changes From Univariate Analysis

Without adjusting for the global value, a negative correlation between absolute CBF and age was observed in the right middle frontal gyrus extending to middle cingulate, left inferior parietal lobule, right middle temporal gyrus, and bilateral thalamus, caudate body, and cerebellum in regression analysis (**Table 2**). Compared to the younger group, absolute regional CBF in bilateral superior frontal gyrus, right middle frontal gyrus, right superior temporal gyrus, and bilateral caudate body and cerebellum was decreased in the older group (Supplementary Table 1). No significant increases in absolute regional CBF with aging were observed in either regression model or group comparison.

**Table 2:** Regions showing a negative correlation between absolute CBF and age.

Structure	BA	*X*	*Y*	*Z*	*Z* max	Size (ml)
Right middle frontal gyrus ^∗^	6	44	8	52	4.45	144.37
Bilateral middle cingulate		0	−33	46	3.58	2.15
Left inferior parietal lobule ^∗^	40	−60	−52	42	4.16	21.13
Right middle temporal gyrus	21	68	−14	−12	3.50	1.18
Left thalamus ^∗^		−4	−18	18	4.08	2.94
Right thalamus ^∗^		9	−18	20	4.49	^∗∗^
Left caudate body ^∗^		−6	12	9	4.26	3.76
Right caudate body ^∗^		8	12	9	4.60	^∗∗^
Left cerebellum (Tuber) ^∗^		−46	−51	−32	4.36	69.30
Right cerebellum (Tuber) ^∗^		50	−58	−32	4.25	^∗∗^
Bilateral cerebellum (Declive) ^∗^		4	−74	−21	4.25	^∗∗^

*BA, Brodmann area. Regression (*P* < 0.001, uncorrected). ^∗^Regions survived family wise error correction at *P* < 0.05. ^∗∗^Region belongs to the cluster for which the size is provided above.*

**FIGURE 1:**
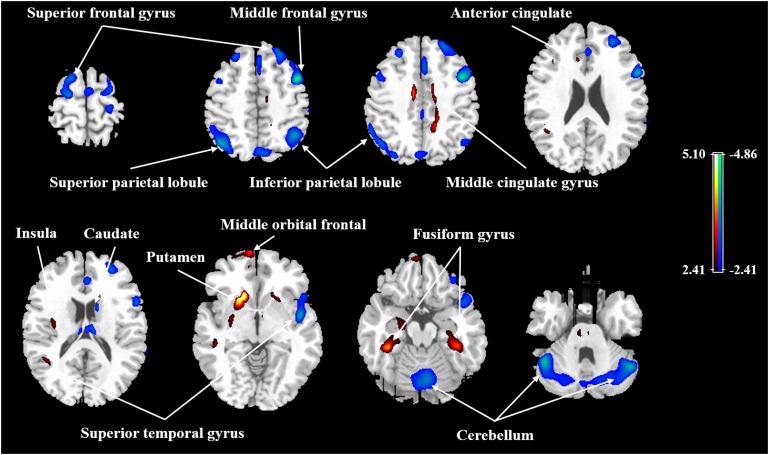
Age-related changes in relative CBF in all participants from regression analysis. Cold color indicates regions in which CBF negatively correlated with age, and warm color indicates regions in which CBF positively correlated with age. A threshold of 2.41 (*P* < 0.01) was used to overlay SPM maps onto a standard MRI brain template.

**FIGURE 2:**
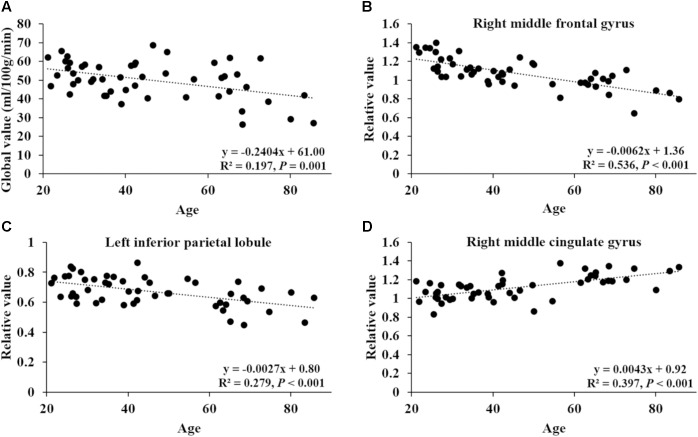
Correlation of age with global CBF value and relative CBF values in three sample regions. **(A)** The correlation between age and global value from the CBF map. **(B–D)** The correlations between age and relative values of right middle frontal gyrus (44, 8, 50), left inferior parietal lobule (–58, –52, 42) and right middle cingulate gyrus (15, –14, 46), obtained *post hoc* within a spherical volume of interest (4 mm radius).

After ANCOVA normalization for the global value, both negative and positive correlations were observed between regional CBF and age (**Table 3** and **Figure 1**). Relative CBF in bilateral superior frontal gyrus, right middle frontal gyrus, left superior parietal lobule, bilateral inferior parietal lobules, right superior temporal gyrus, right caudate body, and bilateral cerebellum was negatively correlated with age. Regions with relatively increased CBF in aging included left anterior cingulate, bilateral middle cingulate gyrus, left insula, left superior temporal gyrus, bilateral fusiform gyrus, bilateral putamen and left middle orbital frontal region. Global value measured from the CBF map negatively correlated with age and gave rise to a rate of decrease at 0.39% per year (**Figure 2A**). Sample plots for the three major regions from the regression analysis, including right middle frontal gyrus, left inferior parietal lobule and right middle cingulate gyrus, are shown in **Figures 2B–D**. In the group comparison, there was a non-significant decline in global CBF values (51.9 ± 7.6 vs. 48.1 ± 11.8 ml/100 g/min; *P* = 0.17) for the younger and older groups. Relative to the younger group, older subjects showed relatively decreased CBF in the left superior frontal gyrus, right middle frontal gyrus, right superior temporal gyrus, right caudate body, and bilateral cerebellum, and relatively increased CBF in the right medial frontal gyrus, left middle temporal gyrus, left middle orbital frontal region, and left putamen (Supplementary Table 2).

**Table 3:** Regions with age-related relative changes in globally adjusted CBF.

Structure	BA	*X*	*Y*	*Z*	*Z* max	Size (ml)
*Negative correlation*						
Left superior frontal gyrus	6	−20	8	69	3.52	3.19
Right superior frontal gyrus	8	24	39	52	3.60	1.99
Right middle frontal gyrus ^∗^	6	44	8	50	4.55	4.48
Left superior parietal lobule	7	−40	−63	52	3.90	1.83
Left inferior parietal lobule	40	−58	−52	42	3.44	0.47
Right inferior parietal lobule	40	40	−54	54	3.88	1.33
Right superior temporal gyrus	22, 38	46	−2	−4	3.81	1.24
Right caudate body		8	12	9	3.86	0.67
Left cerebellum (Tuber)		−46	−52	−30	4.08	2.03
Right cerebellum (Declive/Tuber)		48	−58	−30	3.93	1.73
Bilateral cerebellum (Declive/Culmen)		4	−74	−21	3.48	1.71
*Positive correlation*						
Left anterior cingulate	32	−12	26	24	4.01	0.46
Left middle cingulate gyrus		−12	−9	44	4.28	0.59
Right middle cingulate gyrus ^∗^		15	−14	46	4.78	1.81
Left insula	13	−33	−9	18	3.95	0.39
Left superior temporal gyrus	22	−45	−42	10	3.85	1.07
Left fusiform gyrus ^∗^	37	−39	−36	−16	4.47	0.55
Right fusiform gyrus	20	38	−34	−18	3.71	0.45
Left putamen ^∗^		−18	20	−4	5.09	2.51
Right putamen		21	21	3	3.93	0.41
Left middle orbital frontal	11	−9	70	−6	3.84	1.07

*BA, Brodmann area. Regression (P < 0.001, uncorrected) with ANCOVA normalization for global value. ^∗^Regions survived family wise error correction at *P* < 0.05.*

In our participants, women were on average younger than men (40.8 vs. 52.1 years), and women had 18.3% greater global CBF. This difference in global CBF was significant (*P* = 0.02) after adjusting for age. However, no sex differences were seen in relative CBF values of three sample regions after normalizing for global value (Supplementary Table 3). Apart from the global CBF value in women, the correlations between regional relative CBF values and age were still preserved in women and men (Supplementary Figures 1, 2).

### Age-Related CBF Pattern From Multivariate Analysis

**FIGURE 3:**
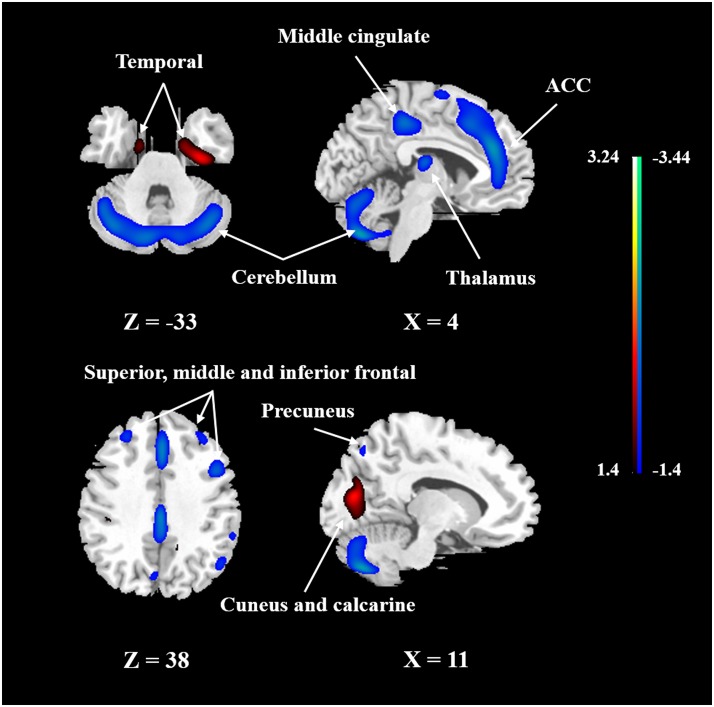
The network of CBF changes with aging identified by SSM/PCA from 50 healthy subjects. Cold color indicates regions loading negatively correlated with age, and warm color indicates regions loading positively correlated with age. The pattern was overlaid onto a standard MRI brain template to display voxels that were reliable at *P* < 0.05 based on the bootstrapping algorithm. ACC, anterior cingulate gyrus.

To identify the CBF pattern associated with healthy aging, we applied SSM/PCA analysis to the MRI CBF maps from the 50 healthy adult subjects. Focusing on the major source of variance in the CBF maps, we initially restricted the multiple regression model to include the subject scores from the first 6 SSM component patterns. The model that included PC1, PC5, and PC6 (variance accounted for = 26.68, 5.38, and 3.68% and regression coefficient β = −0.494, −0.549, and −0.208, respectively) was the best in predicting the effect of age (*F* = 21.927, *P* < 0.001). (The patterns for PC1 alone or PC1 plus PC5 predicting the effect of age were also identified and are presented in Supplementary Figures 3, 4, respectively.) **Figure 3** shows the age-related CBF pattern (total variance accounted for 35.74%) that was reliable at *P* < 0.05 based on the bootstrapping algorithm (1000 iterations). This covariance pattern was characterized by relatively decreased CBF in frontal and parietal areas and cerebellum, along with relatively increased CBF in temporal and occipital areas. The locations of all brain regions and coordinates for voxels with local minima and maxima pattern weights for this age-related pattern are shown in **Table 4**.

**Table 4:** Regions of the age-related CBF pattern.

Structure	BA	*X*	*Y*	*Z*	*T*	Size (ml)
*Negative Loading*						
Left middle frontal gyrus/superior frontal gyrus	8, 9, 46	−27	39	38	2.11	5.21
Right superior frontal gyrus/middle frontal gyrus	9, 11, 46	35	36	38	1.93	7.11
Left middle frontal gyrus	9	−44	11	45	1.64	0.55
Right middle frontal gyrus/inferior frontal gyrus	6, 9, 44	44	11	42	2.45	14.55
Bilateral cingulate gyrus	32	2	20	44	2.85	19.42
Bilateral paracentral lobule/middle cingulate	4, 23	5	−39	69	1.64	8.84
Left superior parietal lobule/inferior parietal lobule	7	−24	−74	47	1.79	1.35
Right superior parietal lobule/inferior parietal lobule	7, 40	33	−63	53	1.88	5.48
Left supramarginal gyrus	40	−60	−45	32	1.65	0.42
Right inferior parietal lobule/superior temporal gyrus/postcentral gyrus	40, 42, 48	57	−42	39	1.90	4.14
Left precuneus	7	−6	−75	42	2.08	1.29
Right precuneus	7	11	−72	50	1.93	0.69
Bilateral thalamus		2	−17	14	2.72	2.55
Left cerebellum (pyramis)		−36	−68	−44	3.35	58.69
Right cerebellum (inferior semi-lunar lobule)		39	−65	−47	4.05	^∗∗^
*Positive loading*						
Right cuneus/calcarine	18	23	−72	6	2.96	44.16
Left superior frontal gyrus	11	−11	69	−9	2.07	5.65
Left orbital gyrus	11	−12	21	−27	2.07	0.70
Left temporal pole/superior temporal gyrus	20, 39, 36	−26	−8	−47	3.51	59.53
Right temporal pole/parahippocampal gyrus	20	29	−8	−47	3.72	18.63

*BA, Brodmann area. The coordinates were reliable at P < 0.05 based on the bootstrapping algorithm. Cluster size > 100. ^∗∗^Region belongs to the cluster for which the size is provided above.*

**FIGURE 4:**
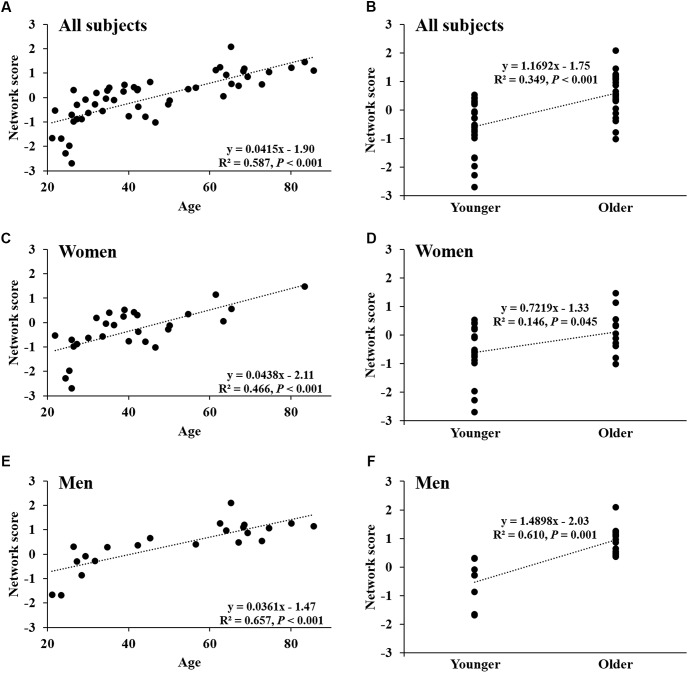
Network score of age-related CBF pattern. **(A)** Network score in relation to age in all healthy participants. **(B)** Differences in network score between older subjects and younger subjects in all healthy participants. **(C,D)** Network score in relation to age, and differences in network score between older subjects and younger subjects in women. **(E,F)** Network score in relation to age, and differences in network score between older subjects and younger subjects in men.

**FIGURE 5:**
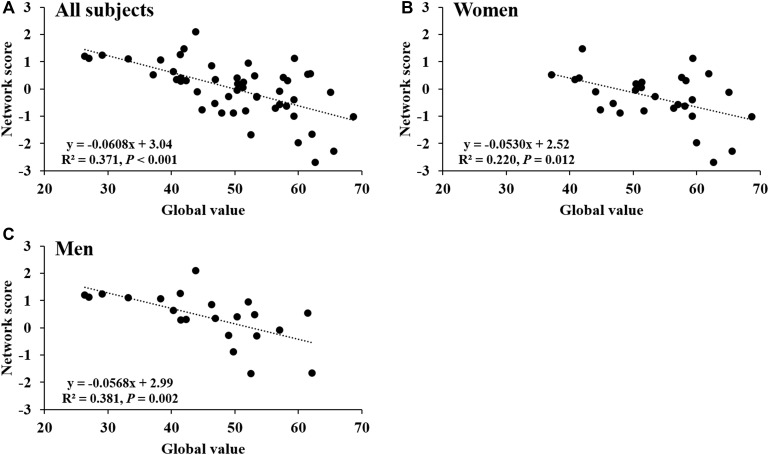
Network score related to global value from CBF map. **(A)** This network score of combined PC1, PC5, and PC6 from PCA/SSM analysis correlated with the global value of CBF map in all healthy participants. **(B)** The network score correlated with the global value of CBF map in female subjects. **(C)** The network score correlated with the global value of CBF map in male subjects.

Network score of this pattern correlated positively and strongly with age (*R*^2^ = 0.59, *P* < 0.001; **Figure 4A**) and was elevated (*P* < 0.001) in the older group compared with the younger group (**Figure 4B**); these relationships were preserved in women (**Figures 4C,D**) and men (**Figures 4E,F**) separately. The network score was also related negatively to global value from the CBF map in all subjects (*R*^2^ = 0.37, *P* < 0.001; **Figure 5A**), in women (*P* = 0.012; **Figure 5B**) and men (*P* = 0.002; **Figure 5C**) separately.

## Discussion

This study demonstrates the full extent of age-related CBF changes measured with pCASL over the full spectrum of healthy aging. Similar to an FDG-PET study analyzed with SPM (Petit-Taboue et al., 1998), absolute CBF value showed significant decline across most parts of the brain, but both relative decreases and increases in regional CBF were observed after adjusting for global value. In our present study, relative CBF in bilateral superior frontal gyrus, right middle frontal gyrus, left superior parietal lobule, bilateral inferior parietal lobules, right superior temporal gyrus, right caudate body and bilateral cerebellum were negatively correlated with age; regions in temporal and orbital frontal lobes, anterior and middle cingulate, and putamen showed relatively increased CBF during aging. We further identified an age-related CBF pattern using SSM/PCA that accounted for more than 35% of the variance, including both negative and positive region weights in CBF.

Aging causes a wide array of anatomical and functional changes to the brain. Age-related CBF decrease has been observed in H_2_^15^O PET studies (Pantano et al., 1984; Leenders et al., 1990), although it could not be always reproduced after partial volume correction (Meltzer et al., 2000). The observation of global CBF reduction with age at about 0.39% per year in this study is consistent with previous ASL studies, which reported CBF decline at about 0.38 ∼ 0.45% per year (Parkes et al., 2004; Biagi et al., 2007; Wagner et al., 2012; Ambarki et al., 2015; De Vis et al., 2015), even after adjusting for brain atrophy in aging (Asllani et al., 2009; Chen et al., 2011). The strength of the correlation between age and global CBF (*R*^2^ = 0.1965) in our study is also very similar to that in a previous H_2_^15^O PET study (*R*^2^ = 0.1936) (Pantano et al., 1984). The global CBF values measured for the younger and older subjects in this study are highly comparable to those reported in an H_2_^15^O PET study with similar age range (Aanerud et al., 2012). While the global CBF of our younger group is more consistent with most previous ASL studies (Chen et al., 2011; De Vis et al., 2015) and H_2_^15^O PET studies (49 ∼ 53 ml/100 g/min) (Pantano et al., 1984), the global CBF of our older group is somewhat higher because of the lower cut-off for group classification by median age (41.8 vs. 55 years). CBF decline may be associated with reduction in neuronal and synaptic number and activity (Parkes et al., 2004), and degeneration in the cerebral microvasculature (Farkas and Luiten, 2001) during aging.

As the current gold standard measurement of CBF, H_2_^15^O PET studies have highlighted decreased CBF in frontal and temporal regions with aging (Pantano et al., 1984; Meltzer et al., 2000). Studies that compared ASL and H_2_^15^O PET have demonstrated the utility of ASL for accurate and reproducible CBF measurements (Fan et al., 2016). In our present study, we observed that age-related CBF decreases in the middle frontal gyrus, inferior parietal lobule, thalamus, caudate and cerebellum, which have been reported in previous ASL studies (Parkes et al., 2004; Lu et al., 2011; Preibisch et al., 2011; Wierenga et al., 2013). Consistent with a previous study (Preibisch et al., 2011), some regions exhibited relatively increased perfusion in aging after correcting for global value, such as middle cingulate gyrus, fusiform gyrus and putamen. These regional relative increases in CBF, which were speculated to be a compensatory response to aging (Preibisch et al., 2011), may be attributed to selective regional vulnerabilities to brain degeneration, with some areas relatively preserved compared to other regions in normal aging. Aside from small sample sizes and different analytic methods, differences in findings of age-related CBF change might also be attributable to vascular risk factors (Birdsill et al., 2013; Bangen et al., 2014), sub-clinical cerebrovascular disease (Crane et al., 2015; Gregg et al., 2015), sedentary behavior (Zlatar et al., 2014), APOE genotype (Rane et al., 2013; Wierenga et al., 2013), and AD family history (Fleisher et al., 2009; Okonkwo et al., 2014).

Our characteristic CBF network derived with SSM/PCA is similar to a metabolic network that also showed decreased frontal and parietal association cortical metabolism in aging, previously derived with FDG-PET based on either region of interest (Moeller et al., 1996) or voxelwise (Zuendorf et al., 2003) analysis. However, the cerebellum showed negative weights in our CBF pattern, but was relatively preserved in those other metabolic patterns. The discrepancy in regional measures of perfusion and metabolism could be attributed to the utilization of different analytical methods. It has also been suggested that residual differences between perfusion and metabolism during healthy aging might be attributable to the use of global values for parameters such as partition coefficient, dispersion and time delay for regional CBF and the lumped constant for regional metabolic rate (Bentourkia et al., 2000).

Sex could be an important confounder in the CBF comparison and derivation of the age-related CBF pattern, although we controlled for it as a covariate in the analysis. As has been previously reported (Liu et al., 2012), we found overall global CBF was increased in women after adjusting for age. Other investigators have reported that premenopausal women exhibited higher CBF than young men, while there was no difference in CBF between older postmenopausal women and older men (Liu et al., 2016). Those findings could be related to differential effects of sex hormones between women and men with aging, for example the effects of estrogen on vascular endothelial growth factor (VEGF). Nonetheless, apart from global CBF in women, we found that the correlations between regional relative CBF values and PCA network score with age were maintained in either women or men separately, indicating that the age-related CBF pattern was relatively independent of sex in our present study.

Our age-related CBF pattern, which mainly features decreases in the anterior part of the brain and cerebellum, is quite different from previously reported Alzheimer’s disease (AD-related) or Parkinson’s disease (PD-related) CBF patterns measured with ASL. In those disease-related patterns, CBF was mainly decreased in the posterior areas of the brain, e.g., posterior cingulate and inferior occipital gyrus in AD (Asllani et al., 2008), and parietal and occipital cortex in PD (Ma et al., 2010; Melzer et al., 2011; Teune et al., 2014). Interestingly, we observed some areas with opposite region weights from disease-related patterns. For instance, cerebellum showed decreased loading in our age-related pattern but increased loading in the PD-related pattern (Teune et al., 2014). Furthermore, taking into account that regional CBF correlated with cognitive function and was sensitive to aerobic exercise or cognitive training in the elderly (Chapman et al., 2013, 2015), this age-related CBF pattern is a promising method for monitoring the aging process and distinguishing neurodegenerative diseases from normal aging. A recent study observed that CBF, especially in the frontal lobe and anterior cingulate cortex, which belongs to the default mode network, predicts cognitive ability in healthy individuals in a 4-year follow-up (De Vis et al., 2018). However, the overlap and distinction between age-related CBF pattern and cognitive predicting CBF pattern should be further explored, since we did not have comprehensive longitudinal cognitive testing for the cohort in this study.

Several regions in the frontal lobes and cerebellum are involved in CBF reduction in our age-related pattern. Some other studies have suggested that the frontal region may be more vulnerable to aging compared to other parts of the brain. An age-related volumetric pattern identified by SSM shared some areas represented in our CBF pattern, such as frontal and parietal regions (Brickman et al., 2007; Bergfield et al., 2010). Microvascular pathology as indicated by white matter hyperintensity predominates in the frontal lobes and preferentially affects glucose metabolism in prefrontal cortex in normal aging (Jagust, 2013). In addition, the cerebellum shows significant loss of neurons and a deficit in synaptic plasticity with age, even earlier than the hippocampus (Woodruff-Pak et al., 2010). However, temporal regions, which are especially involved in AD, showed positive weights (e.g., parahippocampal gyrus) in our age-related CBF pattern. Since potential AD pathology may have been present in some cognitively normal elderly subjects, it may suggest a compensatory effect on CBF regulation in temporal regions before the onset of AD. Although our age-related CBF pattern supports structural and functional changes in aging from previous studies, the mechanism and progression of aging in the brain need further investigation.

### Limitations

Apart from physiological changes, CBF reduction might also be confounded by factors such as increases in partial volume effects caused by brain atrophy and cortical thinning, and increases in arterial transit time in aging. In addition, although we tried to exclude individuals with cerebrovascular disease and neurodegenerative diseases by thorough medical history and structural MRI, underlying risk factors and neuropathology were not assessed in this study, such as APOE genotype and amyloid-β, which have been observed to be related to CBF changes in normal aging (Wierenga et al., 2013; Michels et al., 2016). For instance, a substantial proportion of healthy elderly subjects have brain amyloid deposition, as demonstrated using PET with^11^C Pittsburgh Compound B, especially in APOE ε4 carriers and the oldest old (Rowe et al., 2010). Moreover, this is a cross-sectional study. Since brain reserve and environmental factors may differ between individual subjects, longitudinal studies using data from the same individual may yield more reliable findings in age-related CBF changes. Clinical conversion also occurs in a sufficiently long study, allowing for identification of underlying neuropathology in putatively healthy elderly subjects. Furthermore, although the age range of our participants was from 21 to 85 years, the sample size is still relatively small.

## Conclusion

This is the first study to derive an age-related CBF pattern using ASL MRI with SSM/PCA multivariate analysis. This network, which is distinct from disease-related CBF patterns in AD and PD, is characterized by decreased CBF in frontal and parietal areas and cerebellum, and relatively preserved CBF in temporal, occipital and orbital frontal areas. A larger longitudinal study is needed to validate the characteristic age-related perfusion network as a suitable marker of aging.

## Data Availability Statements

The raw data supporting the conclusions of this manuscript will be made available by the authors, without undue reservation, to any qualified researcher.

## Ethics Statement

This study was carried out in accordance with all applicable Department of Health and Human Services regulations 45 CFR 46, Food and Drug Administration regulations 21 CFR 50, 21 CFR 56, 21 CFR 812, and the Health Insurance Portability and Accountability Act (HIPAA) with the recommendations of the Northwell Health IRB, with written informed consent from all subjects. All subjects gave written informed consent in accordance with the Declaration of Helsinki. The protocol was approved by the Northwell Health IRB.

## Author Contributions

NZ processed and analyzed the imaging data and wrote the manuscript. MG and TG designed the study. MG and DE recruited and assessed the participants. MG, TG, and YM reviewed the manuscript. BC preprocessed some of the imaging data. JG, SP, and YM gave the technical direction for data analysis. PK conducted the MRI procedure and acquired the imaging data. DE gave crucial comments for the manuscript.

## Conflict of Interest Statement

The authors declare that the research was conducted in the absence of any commercial or financial relationships that could be construed as a potential conflict of interest.

## Footnotes

1 http://www.feinsteinneuroscience.org

## References

[B1] Aanerud J. Borghammer P. Chakravarty M. M. Vang K. Rodell A. B. Jonsdottir K. Y. (2012). Brain energy metabolism and blood flow differences in healthy aging. *J. Cereb. Blood Flow Metab.* 32 1177–1187. 10.1038/jcbfm.2012.18jcbfm201218 22373642 PMC3390816

[B2] Alsop D. C. Detre J. A. Golay X. Gunther M. Hendrikse J. Hernandez-Garcia L. (2015). Recommended implementation of arterial spin-labeled perfusion MRI for clinical applications: a consensus of the ISMRM perfusion study group and the European consortium for ASL in dementia. *Magn. Reson. Med.* 73 102–116. 10.1002/mrm.25197 24715426 PMC4190138

[B3] Ambarki K. Wahlin A. Zarrinkoob L. Wirestam R. Petr J. Malm J. (2015). Accuracy of parenchymal cerebral blood flow measurements using pseudocontinuous arterial spin-labeling in healthy volunteers. *AJNR Am. J. Neuroradiol.* 36 1816–1821. 10.3174/ajnr.A4367ajnr.A4367 26251434 PMC7965027

[B4] Asllani I. Habeck C. Borogovac A. Brown T. R. Brickman A. M. Stern Y. (2009). Separating function from structure in perfusion imaging of the aging brain. *Hum. Brain Mapp.* 30 2927–2935. 10.1002/hbm.20719 19172645 PMC2733928

[B5] Asllani I. Habeck C. Scarmeas N. Borogovac A. Brown T. R. Stern Y. (2008). Multivariate and univariate analysis of continuous arterial spin labeling perfusion MRI in Alzheimer’s disease. *J. Cereb. Blood Flow Metab.* 28 725–736. 10.1038/sj.jcbfm.9600570 17960142 PMC2711077

[B6] Bangen K. J. Nation D. A. Clark L. R. Harmell A. L. Wierenga C. E. Dev S. I. (2014). Interactive effects of vascular risk burden and advanced age on cerebral blood flow. *Front. Aging Neurosci.* 6:159. 10.3389/fnagi.2014.00159 25071567 PMC4083452

[B7] Bentourkia M. Bol A. Ivanoiu A. Labar D. Sibomana M. Coppens A. (2000). Comparison of regional cerebral blood flow and glucose metabolism in the normal brain: effect of aging. *J. Neurol. Sci.* 181 19–28. 10.1016/S0022-510X(00)00396-8 11099707

[B8] Bergfield K. L. Hanson K. D. Chen K. Teipel S. J. Hampel H. Rapoport S. I. (2010). Age-related networks of regional covariance in MRI gray matter: reproducible multivariate patterns in healthy aging. *Neuroimage* 49 1750–1759. 10.1016/j.neuroimage.2009.09.051 19796692 PMC2789892

[B9] Biagi L. Abbruzzese A. Bianchi M. C. Alsop D. C. Del Guerra A. Tosetti M. (2007). Age dependence of cerebral perfusion assessed by magnetic resonance continuous arterial spin labeling. *J. Magn. Reson. Imaging* 25 696–702. 10.1002/jmri.20839 17279531

[B10] Birdsill A. C. Carlsson C. M. Willette A. A. Okonkwo O. C. Johnson S. C. Xu G. (2013). Low cerebral blood flow is associated with lower memory function in metabolic syndrome. *Obesity* 21 1313–1320. 10.1002/oby.20170 23687103 PMC3742665

[B11] Brickman A. M. Habeck C. Zarahn E. Flynn J. Stern Y. (2007). Structural MRI covariance patterns associated with normal aging and neuropsychological functioning. *Neurobiol. Aging* 28 284–295. 10.1016/j.neurobiolaging.2005.12.016 16469419

[B12] Chapman S. B. Aslan S. Spence J. S. Defina L. F. Keebler M. W. Didehbani N. (2013). Shorter term aerobic exercise improves brain, cognition, and cardiovascular fitness in aging. *Front. Aging Neurosci.* 5:75. 10.3389/fnagi.2013.00075 24282403 PMC3825180

[B13] Chapman S. B. Aslan S. Spence J. S. Hart JJ Jr Bartz E. K. Didehbani N. (2015). Neural mechanisms of brain plasticity with complex cognitive training in healthy seniors. *Cereb. Cortex* 25 396–405. 10.1093/cercor/bht234bht234 23985135 PMC4351428

[B14] Chen J. J. Rosas H. D. Salat D. H. (2011). Age-associated reductions in cerebral blood flow are independent from regional atrophy. *Neuroimage* 55 468–478. 10.1016/j.neuroimage.2010.12.032S1053-8119(10)01616-2 21167947 PMC3435846

[B15] Crane D. E. Black S. E. Ganda A. Mikulis D. J. Nestor S. M. Donahue M. J. (2015). Gray matter blood flow and volume are reduced in association with white matter hyperintensity lesion burden: a cross-sectional MRI study. *Front. Aging Neurosci.* 7:131. 10.3389/fnagi.2015.00131 26217223 PMC4495336

[B16] De Vis J. B. Hendrikse J. Bhogal A. Adams A. Kappelle L. J. Petersen E. T. (2015). Age-related changes in brain hemodynamics; a calibrated MRI study. *Hum. Brain Mapp.* 36 3973–3987. 10.1002/hbm.22891 26177724 PMC6869092

[B17] De Vis J. B. Peng S. L. Chen X. Li Y. Liu P. Sur S. (2018). Arterial-spin-labeling (ASL) perfusion MRI predicts cognitive function in elderly individuals: A 4-year longitudinal study. *J. Magn. Reson. Imaging.* 10.1002/jmri.25938 [Epub ahead of print]. 29292540 PMC6028323

[B18] Eidelberg D. (2009). Metabolic brain networks in neurodegenerative disorders: a functional imaging approach. *Trends Neurosci.* 32 548–557. 10.1016/j.tins.2009.06.003 19765835 PMC2782537

[B19] Fan A. P. Jahanian H. Holdsworth S. J. Zaharchuk G. (2016). Comparison of cerebral blood flow measurement with [15O]-water positron emission tomography and arterial spin labeling magnetic resonance imaging: a systematic review. *J. Cereb. Blood Flow Metab.* 36 842–861. 10.1177/0271678X166363930271678X16636393 26945019 PMC4853843

[B20] Farkas E. Luiten P. G. (2001). Cerebral microvascular pathology in aging and Alzheimer’s disease. *Prog. Neurobiol.* 64 575–611. 10.1016/S0301-0082(00)00068-X 11311463

[B21] Fleisher A. S. Podraza K. M. Bangen K. J. Taylor C. Sherzai A. Sidhar K. (2009). Cerebral perfusion and oxygenation differences in Alzheimer’s disease risk. *Neurobiol. Aging* 30 1737–1748. 10.1016/j.neurobiolaging.2008.01.012 18325636 PMC2746874

[B22] Gregg N. M. Kim A. E. Gurol M. E. Lopez O. L. Aizenstein H. J. Price J. C. (2015). Incidental cerebral microbleeds cerebral blood flow in elderly individuals. *JAMA Neurol.* 72 1021–1028. 10.1001/jamaneurol.2015.13592388928 26167811 PMC4724412

[B23] Habeck C. Foster N. L. Perneczky R. Kurz A. Alexopoulos P. Koeppe R. A. (2008). Multivariate and univariate neuroimaging biomarkers of Alzheimer’s disease. *Neuroimage* 40 1503–1515. 10.1016/j.neuroimage.2008.01.056S1053-8119(08)00102-X 18343688 PMC2441445

[B24] Jagust W. (2013). Vulnerable neural systems and the borderland of brain aging and neurodegeneration. *Neuron* 77 219–234. 10.1016/j.neuron.2013.01.002 23352159 PMC3558930

[B25] Leenders K. L. Perani D. Lammertsma A. A. Heather J. D. Buckingham P. Healy M. J. (1990). Cerebral blood flow, blood volume and oxygen utilization: normal values and effect of age. *Brain* 113(Pt 1), 27–47. 10.1093/brain/113.1.27 2302536

[B26] Liu W. Lou X. Ma L. (2016). Use of 3D pseudo-continuous arterial spin labeling to characterize sex and age differences in cerebral blood flow. *Neuroradiology* 58 943–948. 10.1007/s00234-016-1713-y 27380039

[B27] Liu Y. Zhu X. Feinberg D. Guenther M. Gregori J. Weiner M. W. (2012). Arterial spin labeling MRI study of age and gender effects on brain perfusion hemodynamics. *Magn. Reson. Med.* 68 912–922. 10.1002/mrm.23286 22139957

[B28] Lu H. Xu F. Rodrigue K. M. Kennedy K. M. Cheng Y. Flicker B. (2011). Alterations in cerebral metabolic rate and blood supply across the adult lifespan. *Cereb. Cortex* 21 1426–1434. 10.1093/cercor/bhq224bhq224 21051551 PMC3097991

[B29] Ma Y. Huang C. Dyke J. P. Pan H. Alsop D. Feigin A. (2010). Parkinson’s disease spatial covariance pattern: noninvasive quantification with perfusion MRI. *J. Cereb. Blood Flow Metab.* 30 505–509. 10.1038/jcbfm.2009.256 20051975 PMC2949137

[B30] Meltzer C. C. Cantwell M. N. Greer P. J. Ben-Eliezer D. Smith G. Frank G. (2000). Does cerebral blood flow decline in healthy aging? A PET study with partial-volume correction. *J. Nucl. Med.* 41 1842–1848. 11079492

[B31] Melzer T. R. Watts R. MacAskill M. R. Pearson J. F. Rueger S. Pitcher T. L. (2011). Arterial spin labelling reveals an abnormal cerebral perfusion pattern in Parkinson’s disease. *Brain* 134(Pt 3), 845–855. 10.1093/brain/awq377awq377 21310726 PMC3105489

[B32] Michels L. Warnock G. Buck A. Macauda G. Leh S. E. Kaelin A. M. (2016). Arterial spin labeling imaging reveals widespread and Abeta-independent reductions in cerebral blood flow in elderly apolipoprotein epsilon-4 carriers. *J. Cereb. Blood Flow Metab.* 36 581–595. 10.1177/0271678X15605847 26661143 PMC4794091

[B33] Moeller J. R. Ishikawa T. Dhawan V. Spetsieris P. Mandel F. Alexander G. E. (1996). The metabolic topography of normal aging. *J. Cereb. Blood Flow Metab.* 16 385–398. 10.1097/00004647-199605000-00005 8621743

[B34] Okonkwo O. C. Xu G. Oh J. M. Dowling N. M. Carlsson C. M. Gallagher C. L. (2014). Cerebral blood flow is diminished in asymptomatic middle-aged adults with maternal history of Alzheimer’s disease. *Cereb. Cortex* 24 978–988. 10.1093/cercor/bhs381bhs381 23236200 PMC3948496

[B35] Pantano P. Baron J. C. Lebrun-Grandie P. Duquesnoy N. Bousser M. G. Comar D. (1984). Regional cerebral blood flow and oxygen consumption in human aging. *Stroke* 15 635–641. 10.1161/01.STR.15.4.635 6611613

[B36] Parkes L. M. Rashid W. Chard D. T. Tofts P. S. (2004). Normal cerebral perfusion measurements using arterial spin labeling: reproducibility, stability, and age and gender effects. *Magn. Reson. Med.* 51 736–743. 10.1002/mrm.20023 15065246

[B37] Petit-Taboue M. C. Landeau B. Desson J. F. Desgranges B. Baron J. C. (1998). Effects of healthy aging on the regional cerebral metabolic rate of glucose assessed with statistical parametric mapping. *Neuroimage* 7 176–184. 10.1006/nimg.1997.0318 9597659

[B38] Preibisch C. Sorg C. Forschler A. Grimmer T. Sax I. Wohlschlager A. M. (2011). Age-related cerebral perfusion changes in the parietal and temporal lobes measured by pulsed arterial spin labeling. *J. Magn. Reson. Imaging* 34 1295–1302. 10.1002/jmri.22788 21953683

[B39] Rane S. Ally B. A. Hussey E. Wilson T. Thornton-Wells T. Gore J. C. (2013). Inverse correspondence between hippocampal perfusion and verbal memory performance in older adults. *Hippocampus* 23 213–220. 10.1002/hipo.22080 23109214 PMC3878078

[B40] Rowe C. C. Ellis K. A. Rimajova M. Bourgeat P. Pike K. E. Jones G. (2010). Amyloid imaging results from the Australian Imaging, Biomarkers and Lifestyle (AIBL) study of aging. *Neurobiol. Aging* 31 1275–1283. 10.1016/j.neurobiolaging.2010.04.007 20472326

[B41] Shaw T. G. Mortel K. F. Meyer J. S. Rogers R. L. Hardenberg J. Cutaia M. M. (1984). Cerebral blood flow changes in benign aging and cerebrovascular disease. *Neurology* 34 855–862. 10.1212/WNL.34.7.855 6539861

[B42] Takahashi K. Yamaguchi S. Kobayashi S. Yamamoto Y. (2005). Effects of aging on regional cerebral blood flow assessed by using technetium Tc 99m hexamethylpropyleneamine oxime single-photon emission tomography with 3D stereotactic surface projection analysis. *AJNR Am. J. Neuroradiol.* 26 2005–2009. 16155150 PMC8148830

[B43] Teune L. K. Renken R. J. de Jong B. M. Willemsen A. T. van Osch M. J. Roerdink J. B. (2014). Parkinson’s disease-related perfusion and glucose metabolic brain patterns identified with PCASL-MRI and FDG-PET imaging. *Neuroimage Clin.* 5 240–244. 10.1016/j.nicl.2014.06.007 25068113 PMC4110884

[B44] Wagner M. Jurcoane A. Volz S. Magerkurth J. Zanella F. E. Neumann-Haefelin T. (2012). Age-related changes of cerebral autoregulation: new insights with quantitative T2′-mapping and pulsed arterial spin-labeling MR imaging. *AJNR Am. J. Neuroradiol.* 33 2081–2087. 10.3174/ajnr.A3138ajnr.A3138 22700750 PMC7965581

[B45] Wierenga C. E. Clark L. R. Dev S. I. Shin D. D. Jurick S. M. Rissman R. A. (2013). Interaction of age and APOE genotype on cerebral blood flow at rest. *J. Alzheimers Dis.* 34 921–935. 10.3233/JAD-121897WV08W26393U62134 23302659 PMC4124882

[B46] Wintermark M. Sesay M. Barbier E. Borbely K. Dillon W. P. Eastwood J. D. (2005). Comparative overview of brain perfusion imaging techniques. *Stroke* 36 e83–e99. 10.1161/01.STR.0000177839.03321.25 16100027

[B47] Woodruff-Pak D. S. Foy M. R. Akopian G. G. Lee K. H. Zach J. Nguyen K. P. (2010). Differential effects and rates of normal aging in cerebellum and hippocampus. *Proc. Natl. Acad. Sci. U.S.A.* 107 1624–1629. 10.1073/pnas.09142071070914207107 20080589 PMC2824421

[B48] Zhang N. Gordon M. L. Goldberg T. E. (2017). Cerebral blood flow measured by arterial spin labeling MRI at resting state in normal aging and Alzheimer’s disease. *Neurosci. Biobehav. Rev.* 72 168–175. 10.1016/j.neubiorev.2016.11.023 27908711

[B49] Zlatar Z. Z. Wierenga C. E. Bangen K. J. Liu T. T. Jak A. J. (2014). Increased hippocampal blood flow in sedentary older adults at genetic risk for Alzheimer’s disease. *J. Alzheimers Dis.* 41 809–817. 10.3233/JAD-1322529311J2657G6WHR74 24685629 PMC4259215

[B50] Zuendorf G. Kerrouche N. Herholz K. Baron J. C. (2003). Efficient principal component analysis for multivariate 3D voxel-based mapping of brain functional imaging data sets as applied to FDG-PET and normal aging. *Hum. Brain Mapp.* 18 13–21. 10.1002/hbm.10069 12454908 PMC6872041

