# Supplementary material for: The Age-Related Perfusion Pattern Measured With Arterial Spin Labeling MRI in Healthy Subjects
Source: Front Aging Neurosci. 2018 Jul 17;10:214. doi: 10.3389/fnagi.2018.00214 (PMC6056623; doi:10.3389/fnagi.2018.00214)
Supplement: Supplementary file 1 [file Image_1.PDF]

The age-related perfusion pattern measured with arterial spin labeling MRI in healthy subjects

Nan Zhang, Marc L. Gordon*, Yilong Ma, Bradley Chi, Jesus J Gomar, Shichun Peng, Peter B. Kingsley, David Eidelberg, Terry E. Goldberg

* **Correspondence:** Marc L. Gordon: mlgordon@northwell.edu

Supplementary Data

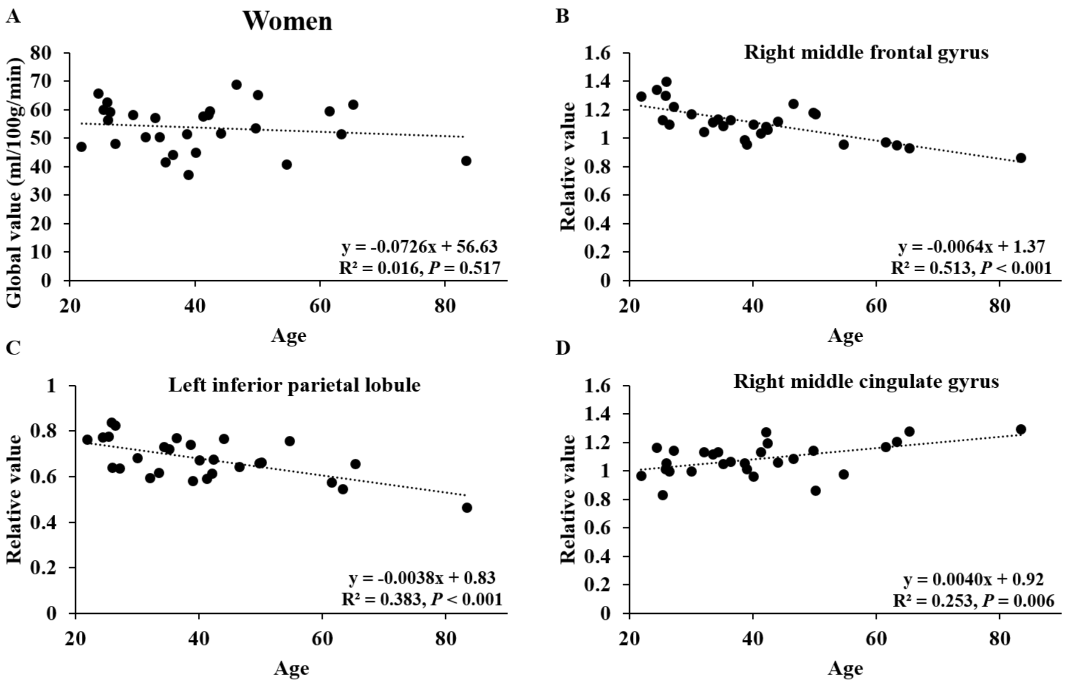

Supplementary Figure 1. Correlation of age with global CBF value and relative CBF values in three sample regions in female subjects. (**A**) The correlation between age and global value from the CBF map. (**B, C, D**) The correlations between age and relative values of right middle frontal gyrus, left inferior parietal lobule and right middle cingulate gyrus. There is no significant correlation between age and global CBF value in female subjects (*P* > 0.05).
