# Supplementary material for: The Age-Related Perfusion Pattern Measured With Arterial Spin Labeling MRI in Healthy Subjects
Source: Front Aging Neurosci. 2018 Jul 17;10:214. doi: 10.3389/fnagi.2018.00214 (PMC6056623; doi:10.3389/fnagi.2018.00214)
Supplement: Supplementary file 2 [file Image_2.PDF]

The age-related perfusion pattern measured with arterial spin labeling MRI in healthy subjects

Nan Zhang, Marc L. Gordon*, Yilong Ma, Bradley Chi, Jesus J Gomar, Shichun Peng, Peter B. Kingsley, David Eidelberg, Terry E. Goldberg

* **Correspondence:** Marc L. Gordon: mlgordon@northwell.edu

Supplementary Data

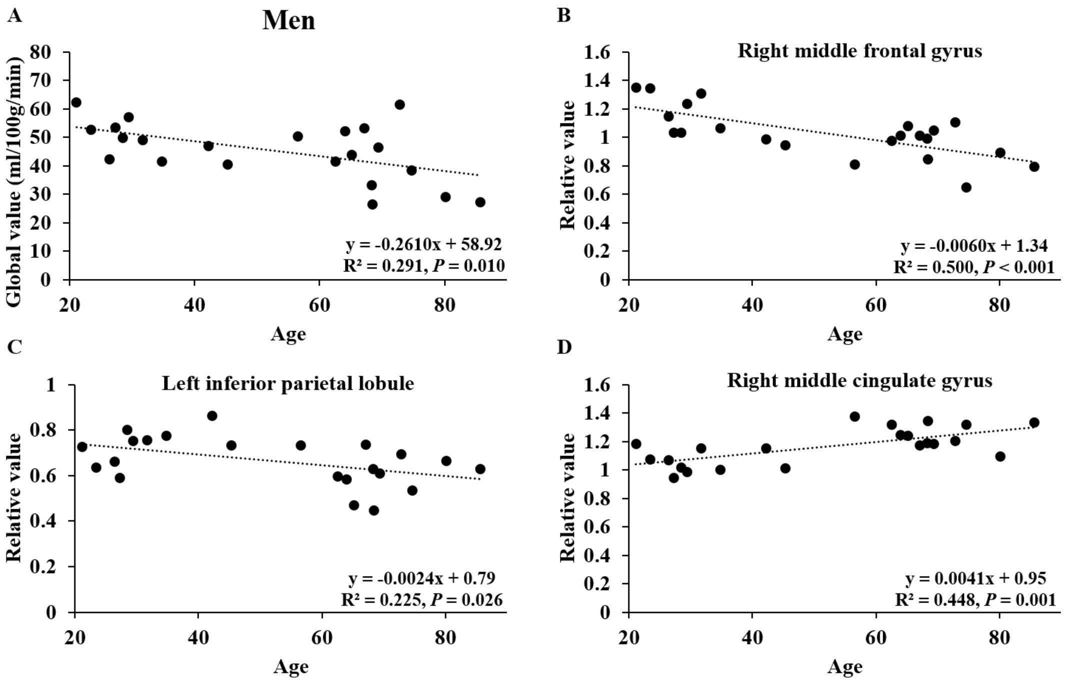

Supplementary Figure 2. Correlation of age with global CBF value and relative CBF values in three sample regions in male subjects. (**A**) The correlation between age and global value from the CBF map. (**B, C, D**) The correlations between age and relative values of right middle frontal gyrus, left inferior parietal lobule and right middle cingulate gyrus.
