# Supplementary material for: The Age-Related Perfusion Pattern Measured With Arterial Spin Labeling MRI in Healthy Subjects
Source: Front Aging Neurosci. 2018 Jul 17;10:214. doi: 10.3389/fnagi.2018.00214 (PMC6056623; doi:10.3389/fnagi.2018.00214)
Supplement: Supplementary file 4 [file Image_4.PDF]

The age-related perfusion pattern measured with arterial spin labeling MRI in healthy subjects

Nan Zhang, Marc L. Gordon*, Yilong Ma, Bradley Chi, Jesus J Gomar, Shichun Peng, Peter B. Kingsley, David Eidelberg, Terry E. Goldberg

*** Correspondence:** Marc L. Gordon: mlgordon@northwell.edu

Supplementary Data

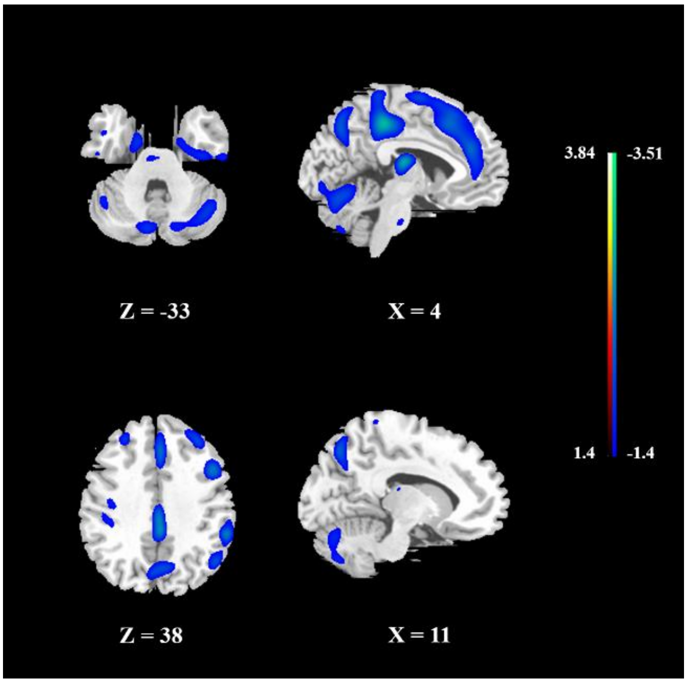

Supplementary Figure 4. The pattern of CBF changes with aging identified through PC1 plus PC5 using SSM/PCA. Cold color indicates regions loading negatively correlated with age.
