# Supplementary material for: The Age-Related Perfusion Pattern Measured With Arterial Spin Labeling MRI in Healthy Subjects
Source: Front Aging Neurosci. 2018 Jul 17;10:214. doi: 10.3389/fnagi.2018.00214 (PMC6056623; doi:10.3389/fnagi.2018.00214)
Supplement: Supplementary file 5 [file Table_1.DOCX]

**The age-related perfusion pattern measured with arterial spin labeling MRI in healthy subjects**

**Nan Zhang****,** **Marc L. Gordon*, Yilong Ma, Bradley Chi, Jesus J Gomar, Shichun Peng, Peter B. Kingsley, David Eidelberg,** **Terry E. Goldberg**

*** Correspondence:** Marc L. Gordon: mlgordon@northwell.edu

**Supplementary Data**

**Supplementary Table 1.** Regions with reduction in absolute CBF in older group compared with younger group

| Structure | BA | X | Y | Z | Z max | Size (ml) |
| --- | --- | --- | --- | --- | --- | --- |
| Left Superior Frontal Gyrus | 6 | -16 | 9 | 72 | 3.10 | 5.09 |
| Right Superior Frontal Gyrus | 6 | 27 | -4 | 69 | 2.53 | 0.81 |
| Right Superior Frontal Gyrus | 6 | 21 | 28 | 62 | 2.68 | 2.26 |
| Right Middle Frontal Gyrus | 6 | 44 | 8 | 58 | 2.75 | 0.75 |
| Right Superior Temporal Gyrus | 22 | 45 | -2 | -6 | 2.72 | 1.95 |
| Left Caudate Body |  | -9 | 4 | 16 | 2.53 | 0.30 |
| Right Caudate Body |  | 9 | 9 | 14 | 2.97 | 0.69 |
| Bilateral Cerebellum (Declive of Vermis) |  | 2 | -78 | -22 | 3.04 | 6.98 |
| Left Cerebellum (Culmen/Declive) |  | -48 | -46 | -34 | 2.80 | 2.27 |

BA = Brodmann area.

Two sample t-test (*P* < 0.001, uncorrected).
