# Supplementary material for: The Age-Related Perfusion Pattern Measured With Arterial Spin Labeling MRI in Healthy Subjects
Source: Front Aging Neurosci. 2018 Jul 17;10:214. doi: 10.3389/fnagi.2018.00214 (PMC6056623; doi:10.3389/fnagi.2018.00214)
Supplement: Supplementary file 6 [file Table_2.DOCX]

**The age-related perfusion pattern measured with arterial spin labeling MRI in healthy subjects**

**Nan Zhang****,** **Marc L. Gordon*, Yilong Ma, Bradley Chi, Jesus J Gomar, Shichun Peng, Peter B. Kingsley, David Eidelberg,** **Terry E. Goldberg**

*** Correspondence:** Marc L. Gordon: mlgordon@northwell.edu

**Supplementary Data**

**Supplementary Table 2.** Regions with relative differences in globally adjusted CBF between older group and younger group

| Structure | BA | X | Y | Z | Z max | Size  (ml) |
| --- | --- | --- | --- | --- | --- | --- |
| *Older < Younger* |  |  |  |  |  |  |
| Left Superior Frontal Gyrus | 6 | -18 | 9 | 69 | 3.96 | 3.61 |
| Right Middle Frontal Gyrus | 6 | 44 | 8 | 51 | 3.89 | 1.36 |
| Right Superior Temporal Gyrus | 22, 38 | 45 | -3 | -4 | 3.71 | 1.77 |
| Right Caudate Body |  | 9 | 9 | 14 | 3.41 | 0.23 |
| Left Cerebellum (Tuber/Declive) |  | -48 | -51 | -30 | 3.76 | 1.02 |
| Right Cerebellum (Declive) |  | 50 | -58 | -30 | 3.45 | 0.25 |
| Bilateral Cerebellum (Declive of Vermis) |  | 2 | -76 | -22 | 3.61 | 1.20 |
| *Older > Younger* |  |  |  |  |  |  |
| Right Medial Frontal Gyrus | 6 | 10 | -14 | 54 | 3.76 | 0.41 |
| Left Middle Temporal Gyrus * | 21 | -44 | -40 | 2 | 4.51 | 1.10 |
| Left Middle Orbital Frontal * | 11 | -9 | 70 | -4 | 3.64 | 0.78 |
| Left Putamen |  | -21 | 15 | -8 | 3.67 | 1.04 |
| Left Putamen |  | -30 | -6 | 0 | 3.21 | 0.40 |

BA = Brodmann area.

Two sample t-test (*P* < 0.001, uncorrected) with ANCOVA normalization for global value.

* Regions survived family-wise error correction at *P* < 0.05.
