# Supplementary material for: The Age-Related Perfusion Pattern Measured With Arterial Spin Labeling MRI in Healthy Subjects
Source: Front Aging Neurosci. 2018 Jul 17;10:214. doi: 10.3389/fnagi.2018.00214 (PMC6056623; doi:10.3389/fnagi.2018.00214)
Supplement: Supplementary file 7 [file Table_3.DOCX]

**The age-related perfusion pattern measured with arterial spin labeling MRI in healthy subjects**

**Nan Zhang****,** **Marc L. Gordon*, Yilong Ma, Bradley Chi, Jesus J Gomar, Shichun Peng, Peter B. Kingsley, David Eidelberg,** **Terry E. Goldberg**

*** Correspondence:** Marc L. Gordon: mlgordon@northwell.edu

**Supplementary Data**

**Supplementary Table 3.** Comparison of global and representative regional CBF values and PCA network score between female subjects and male subjects

|  | Female subjects | Male subjects | *P* |
| --- | --- | --- | --- |
| Global CBF value (ml/100g/min) | 53.66 (8.32) | 45.34 (10.20) | **0.02** |
| Relative value in right middle frontal gyrus | 1.11 (0.13) | 1.03 (0.18) | 0.76 |
| Relative value in left inferior parietal lobule | 0.68 (0.09) | 0.66 (0.10) | 0.43 |
| Relative value in right middle cingulate gyrus | 1.08 (0.12) | 1.16 (0.13) | 0.25 |
| PCA network score | -0.32 (0.94) | 0.41 (0.94) | 0.13 |

Data are provided as mean (SD).

General linear model was used to test the differences between female and male subjects, with age as a covariate.
